# Elucidating the Molecular Mechanisms for the Interaction of Water with Polyethylene Glycol-Based Hydrogels: Influence of Ionic Strength and Gel Network Structure

**DOI:** 10.3390/polym13060845

**Published:** 2021-03-10

**Authors:** Xin Yang, Bronwin L. Dargaville, Dietmar W. Hutmacher

**Affiliations:** Centre for Transformative Biomimetics in Bioengineering, Queensland University of Technology, 60 Musk Avenue, Kelvin Grove, QLD 4059, Australia; x48.yang@hdr.qut.edu.au (X.Y.); bronwin.dargaville@qut.edu.au (B.L.D.)

**Keywords:** hydrogel, swelling, water, poly(ethylene) glycol-diacrylate (PEGDA), ionic strength, biomaterial

## Abstract

The interaction of water within synthetic and natural hydrogel systems is of fundamental importance in biomaterial science. A systematic study is presented on the swelling behavior and states of water for a polyethylene glycol-diacrylate (PEGDA)-based model neutral hydrogel system that goes beyond previous studies reported in the literature. Hydrogels with different network structures are crosslinked and swollen in different combinations of water and phosphate-buffered saline (PBS). Network variables, polyethylene glycol (PEG) molecular weight (MW), and weight fraction are positively correlated with swelling ratio, while “non-freezable bound water” content decreases with PEG MW. The presence of ions has the greatest influence on equilibrium water and “freezable” and “non-freezable” water, with all hydrogel formulations showing a decreased swelling ratio and increased bound water as ionic strength increases. Similarly, the number of “non-freezable bound water” molecules, calculated from DSC data, is greatest—up to six molecules per PEG repeat unit—for gels swollen in PBS. Fundamentally, the balance of osmotic pressure and non-covalent bonding is a major factor within the molecular structure of the hydrogel system. The proposed model explains the dynamic interaction of water within hydrogels in an osmotic environment. This study will point toward a better understanding of the molecular nature of the water interface in hydrogels.

## 1. Introduction

Water is the most abundant, and arguably the most critical component of biological systems. Interactions of matter with water are fundamentally important in determining the physiochemical and biological responses [1,2]. A hydrogel’s three-dimensional (3D) network is built from a hydrophilic polymer that can swell in water and hence maintain a significant volume of water while conserving its structural architecture due to chemical or physical crosslinking of the macromolecular chains. Hydrogels (Figure S1A) have widespread applications in many fields such as biomaterials, drug delivery, tissue engineering, soft robotics, soft electronics and sensors [3,4,5,6,7,8,9]. Hydrogels can be classified in a number of ways, according to their source, composition, and properties [10]. The polymer source is either natural or synthetic. Natural hydrogels used in biomedical applications include agarose, alginate, chitosan, collagen, fibrin, gelatin, hyaluronic acid (HA) and various combinations and derivatives thereof [11,12]. Commonly used synthetic hydrogels include poly(ethylene oxide) (PEO), poly(vinyl alcohol) (PVA), poly(propylene furmarate-*co*-ethylene glycol) (P[PF-*co*-EG]), and synthetic polypeptides as well as many different co-polymers [13].

Hydrogel swelling properties are rooted in the high thermodynamic affinity that this class of materials has for the solvent itself. The physical and interfacial properties are correlated with the configuration of water on the surface and within the hydrogel network [14]. In turn, this organization is dependent on the properties of the surrounding medium. From analyzing the literature, it can be reasoned that detailed systematic studies on the dynamic interaction of water with hydrogel macromolecules are warranted to enhance understanding of the underlying physiochemical mechanisms reported in the hydrogel literature. In many publications, the material–water interface of hydrogels in general is not well defined [2]. Fundamentally, it is known how water behaves: in bulk water, the molecules exist in a dynamic state of interaction. The oxygen atom supplies a partial negative charge due to its strong electronegativity, while the hydrogen atoms carry a partial positive charge. These opposing charges facilitate a type of secondary non-covalent interaction known as hydrogen bonding. An oxygen atom can hydrogen bond to two hydrogen atoms by sharing one of its two lone pairs of electrons with each. Hydrogen bonds are weaker than covalent bonds but strong compared to most other types of secondary bonding. Hydrogen bonding also occurs between water and other species such as solutes, solid surfaces, and hydrophilic polymers. The introduction of these external factors disturbs the hydrogen bonding arrangement in water and causes reorganization, which aims to minimize the energy of the system. It is this precise organization of water that determines many of the physiochemical and biochemical properties of the hydrogel.

Most experimental evidence suggests that the organization of water at a surface is changed to a more highly ordered state, compared to bulk water, for a thickness of 1–4 water molecules, above which it is indistinguishable from bulk water [1]. Three different statuses of water in hydrogel systems are identified and can be designated according to their mobility or their ability to crystallize [15,16,17]. (1) Bound water is strongly bound to polar groups on the macromolecules; subsequently, it has low mobility and is classified as non-freezable due to its inability to crystallize, even at temperatures as low as −100 °C. It is due primarily to direct hydrogen bonding of the water molecules with the macromolecular chains. (2) Intermediate water is more weakly bound to the macromolecules than bound water and consequently has higher mobility and is freezable, albeit at temperatures below 0 °C. Intermediate water is due to both direct interaction with the polymer chains and interaction via other bound water molecules. (3) Free water does not directly interact with the polymer chains and is thus highly mobile and very similar to bulk water in its behavior, freezing at around 0 °C.

The distribution of water into these three states is dependent on the structure of the polymer, in particular the polarity of its functional groups and the spatial arrangement of these groups. A number of different techniques have been used to study these water states: differential scanning calorimetry (DSC) [16,17,18,19,20], nuclear magnetic resonance [21], infrared spectroscopy (IR) [22], and X-ray diffraction [18]. DSC is widely used because it allows quantitative analysis of all three types of water by investigation of their phase transitions. Both the macroscopic and molecular level swelling behavior of neutral hydrogels has not been systematically studied in the biomaterial literature. In particular, only a few studies to date have investigated the influence of the ionic concentration of the medium on the swelling behavior of neutral hydrogels [23]. This is an important variable, as the ionic strength is known to vary with different biological environments [24].

Reviewing the literature, one concludes that polyethylene glycol (PEG) is the most extensively studied synthetic polymer for hydrogel development and application (Figure S1B). The hydrophilic polyether, with a repeat unit of –CH_2_CH_2_O–, has low toxicity and numerous biomedical applications [5,6,25,26,27,28,29]. Insoluble hydrogel networks can be generated by crosslinking PEG end-capped with acrylate groups (PEG-diacrylate (PEGDA)). The physicochemical properties of the resulting gels, including degradation rate, mechanical properties, crosslinking density, and swelling ratio, can be tightly controlled and systematically modified [13,30,31,32,33,34,35].

Figure S1B shows the number of published papers on swelling-related phenomena of PEGDA hydrogels since 1993. Most of the papers focus on swelling in water or phosphate-buffered saline (PBS). Surprisingly, only a limited number of studies have addressed both the swelling behavior and detailed molecular organization of water for PEGDA-based hydrogels [23,36]. In contrast, the interaction of linear PEG with water has been more thoroughly studied [37,38,39], and this body of knowledge forms the basis for the present study on the water content of PEG hydrogels. Specifically, the research reported here investigates the macroscopic swelling behavior and molecular organization of water in crosslinked PEGDA as a model charge-neutral hydrogel with variable network structure and under different hydration conditions. With a view toward biomaterial science, this research helps elucidate precise physiochemical mechanisms underlying the biocompatibility of PEGDA hydrogels.

## 2. Materials and Methods

### 2.1. Materials

Poly(ethylene glycol)-diacrylate (M_n_ = 10,000 Da) and poly(ethylene glycol)-diacrylate (M_n_ = 20,000 Da) were purchased from Laysan Bio Inc. (Arab, AL, USA). Standard phosphate-buffered saline (PBS) tablets were obtained from Oxoid Limited (Basingstoke, Hampshire, UK). LAP (Lithium phenyl-2,4,6-trimethylbenzoylphosphinate) was purchased from Sigma-Aldrich (St. Louis, MO, USA). Ultrapure water, with a resistivity of 18.2 MΩ⋅cm and pH = 7.12, was delivered by Arium^®^ Pro Application-Oriented Ultrapure Water System (Sartorius AG, Goettingen, Germany). All chemicals and materials were used as received, without further purification.

### 2.2. PEGDA Hydrogel Preparation

Photoinitiator solutions were prepared by dissolving 0.5% *w*/*v* LAP in both ultrapure water (pH = 7.12) and 1X PBS (pH = 7.35). PEGDA with different molecular weight (10 or 20 kiloDaltons) (10 or 20k) were dissolved separately in the prepared LAP photoinitiator solutions at the concentration of 10, 15, 20, and 30% *w*/*v*. The mixture was stirred at room temperature and then placed in a 37 °C water bath until homogeneous. The precursor solution of each group (Table S1) was transferred into a Teflon mold consisting of wells (diameter 5.0 mm and height 1.55 mm) and irradiated at a 405 nm in a custom-made (Gelomics, Brisbane, Australia) light emitting diode (LED) crosslinker (20W LED panel) for 5 min.

### 2.3. Dynamic Gravimetric Swelling Ratio

The gravimetric swelling ratio is defined as the fractional increase in the weight of the hydrogel due to water absorption [40]. Five replicates of each group were weighed immediately after crosslinking and then were submerged in ultrapure water or PBS and incubated statically in an oven at 37 °C. The weight of the hydrogels was measured after 5 min, 10 min, 30 min, 1 h, 2 h, 3 h, 1 day, 3 days, and 7 days. Then, the gravimetric swelling ratio of each hydrogel was determined by the following equation:

(1) $$\mathrm{Gravimetric}\;\mathrm{swelling}\;\mathrm{ratio}=\frac{\mathrm{Ws}-\mathrm{Wi}}{\mathrm{Wi}}$$

where *Ws* is the weight of the swollen hydrogel or hydrogel composite at each time point in PBS or ultrapure water, and *Wi* is the weight of the hydrogel immediately after crosslinking.

### 2.4. Dynamic Volumetric Swelling Ratio

The volumetric swelling ratio is defined as the volume increase of the hydrogel due to water absorption. The same samples were used as for the gravimetric swelling ratio test. Five replicates were measured by optical microscope (diameter x height), (Nikon MSZ25, Tokyo, Japan) immediately after crosslinking and weighing and then were submerged in ultrapure water or PBS and incubated statically in an oven at 37 °C. The volume of the hydrogels was measured after 5 min, 10 min, 30 min, 1 h, 2 h, 3 h, 1 day, 3 days, and 7 days. Then, the volumetric swelling ratio of each hydrogel was determined by the following equation:

(2) $$Volumetric\;swelling\;ratio=\frac{Vs-Vi}{Vi}$$

where *Vs* is the volume of hydrogel at each time point after swelling in 1x PBS or ultrapure water, and *Vi* is the volume of hydrogel immediately after crosslinking.

### 2.5. Thermal Analysis and Water Content Determination

DSC was conducted on a TA Instruments Q100 (New Castle, DE, USA) modulated differential scanning calorimeter under nitrogen, applying a heating/cooling cycle [41]. Each swollen PEGDA hydrogel sample was weighed (2–7 mg) and sealed in a T-zero hermetic pan. DSC was performed on all compositions with 3 replicates for each group, to investigate the transition temperatures and content of different water states. The same heating and cooling rate (10 °C/min) and temperature range (−40 to 80 °C) were used for all runs.

The content of freezable water (*Wf*) and non-freezable water (*Wnf*) could be calculated from the DSC thermogram using Equations (3), (4), and (5) [15]:

(3) $$Wf\left(\%\right)=\frac{Qm}{\Delta \mathrm{Hm}}\times 100$$

(4) $$EWC\;\left(wt\%\right)=\frac{Wwet-Wdry}{Wwet}\;\times \;100$$

(5) $$Wnf\left(\%\right)=EWC-Wf$$

(6) $$WRnf=\;\frac{Wnf\left(\%\right)}{Wp\left(\%\right)}$$

(7) $$Nnf=WRnf\;\frac{Mp}{Mw}$$

where *Qm* is the sum of the enthalpy change of all melting endotherms, which is obtained from the integral of the peaks in the thermogram (Figure S3). ΔHm represents the enthalpy of bulk water (334 J⋅g^−1^) [42]. EWC is water content at equilibrium. *Wwet* is the weight of the sample at equilibrium swelling, and *Wdry* is the weight of the same sample after being freeze-dried [15]. *Wf* (%), *Wnf* (%), and *Wp* (%) are the weight percentages of non-freezable bound water, freezable water, and polymer (PEGDA), respectively. The number of non-freezable water molecules per PEG repeat unit (*Nnf*) was calculated using Equations (6) and (7). *WRnf* is the weight ratio of non-freezable bound water. *Mp* and *Mw* are the molecular weight of polymer and water, respectively.

### 2.6. Statistical Analysis

Statistical analysis was conducted with GraphPad Prism 8.2.1 (La Jolla, CA, USA). Statistical significances for all results were determined by one-way ANOVA or two-way ANOVA. Bonferroni post hoc tests were performed whereby *p* < 0.05 was considered to be statistically significant. Data were represented as average ± standard error (* *p* < 0.05, ** *p* < 0.01, *** *p* < 0.001, and **** *p* < 0.0001).

## 3. Results and Discussion

### 3.1. Dynamic Swelling Studies

Based on their material composition and crosslinking chemistry, hydrogels can soak up small or large amounts of water in relationship to their dry weight. Therefore, water can be described as an absorbed liquid that behaves as a discriminating filter in hydrogels to enable the diffusion especially of solute ions but also larger molecules, while the polymer network serves as a structure to maintain the liquid in the construct. Initially, water molecules arriving in the macromolecular network will hydrate the polar, hydrophilic groups, resulting in what is defined as primary bound water. Firstly, as the result of the hydration of the most polar groups in the original network structure, the macromolecular architecture expands and opens up hydrophobic groups. Secondly, then, a so-called hydrophobically-bound water (secondary bound water) interface is arranged in the hydrogel. Most studies in the literature combine the measurements of primary and secondary bound water and then report the result as the total bound water. Thirdly, often neglected in the literature is the dependence of the solvent medium that the hydrogel is made of and/or exposed to. Osmotic pressure is the pressure initiated by water at dissimilar concentrations caused by the dilution of water by dissolved molecules. The driving force for water to penetrate will remain so long as there is a concentration difference between the gel–liquid phase and the surrounding water. Theoretically, there will always be a concentration difference between hydrogels and the medium because the surrounding water can be defined as infinite in magnitude. Hence, there is an osmotic driving force in the direction of the hydrogel due to the interaction of the bound water molecules and the macromolecular chains. Fourthly, the hydrogel can still take up more water and swell after the hydrophobic, polar, and ionic groups are packed with primary and secondary bound water. This water occupies the interim between the macromolecules as well as the volume of micro– and macropores.

Non-ionic neutral hydrogels reach equilibrium swelling at the time the osmotic pressure of the liquid medium is balanced with the sub-chain stretching energy of the polymer network. These physical constraints of the network prevent continuous expansive swelling that would otherwise occur due to the osmotic pressure alone. Swelling behavior can also be influenced by many factors such as pH, ionic strength of the surrounding medium, and temperature [43]. The present work evaluates the influence of ionic strength, polymer molecular weight prior to crosslinking, and the initial polymer weight fraction on the swelling properties of neutral PEGDA hydrogels.

**Figure 1:**
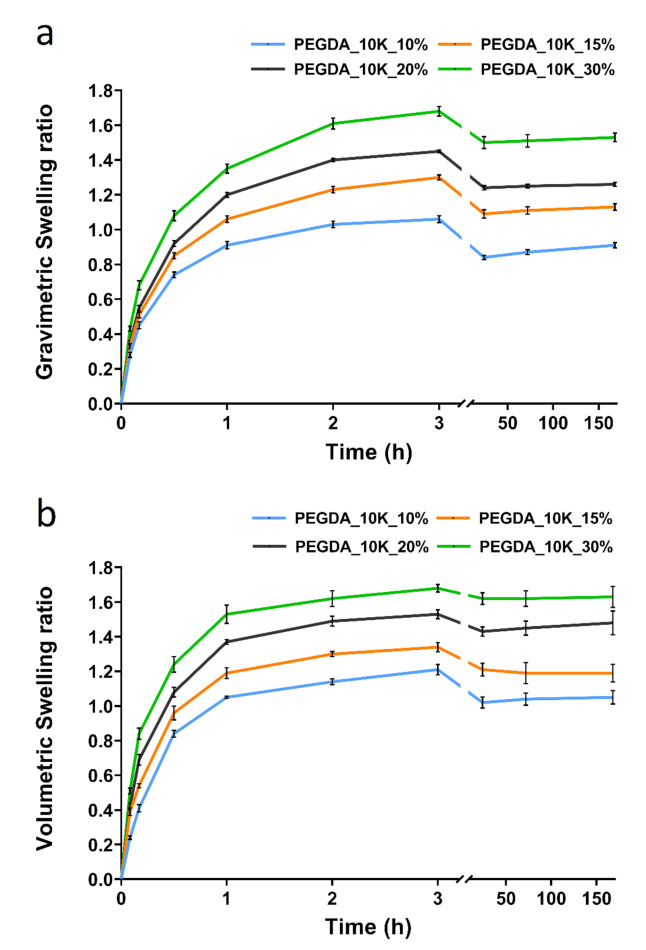
(**a**) Gravimetric equilibrium swelling ratio, and (**b**) Volumetric equilibrium swelling ratio for different weight fraction of polyethylene glycol-diacrylate (PEGDA) 10k crosslinked in water and swollen in PBS (*n* = 5).

To address the knowledge gap of the transformation in hydrogel properties when exposed in water with different ion concentrations, the dynamic swelling ratio of the crosslinked hydrogels swollen in ultrapure water or PBS were compared (Figure S2). This allowed the study of the mechanism of water and ion diffusion into the hydrogels. PEGDA with a molecular weight (*MW*) of 10,000 Daltons (10 k) and with different polymer weight fractions, crosslinked in water and swollen in PBS, was chosen here to demonstrate the gravimetric and volumetric dynamic swelling ratio, as shown in Figure 1. The swelling ratio of PEGDA with a MW of 20,000 Daltons (20 k) followed the same trend as PEGDA 10k. Both dynamic gravimetric and volumetric swelling ratios suggested the hydrogels with PEGDA weight fraction from 10 to 30% swollen at a high rate in the first hour and slowed down over the next two hours. The slight decrease in the swelling ratio from 3 to 24 h was due to diffusion of the soluble non-crosslinked fraction (11.4% ± 0.6%) into the swelling media. The swelling ratio was stabilized after 24 h and reached equilibrium swelling by day 7 (168 h).

The volumetric swelling ratio was not proportional to the gravimetric swelling ratio. It is hypothesized that this was due to the physical spatial constraints imposed by the crosslinking of the polymer macromolecular chains. The volumetric swelling ratio dataset has a larger error bar than the gravimetric swelling ratio data due to the subjective nature of the measurements. The volume of hydrogel at each specific time point was calculated by multiplying the height with the circular upper surface area of each hydrogel disk sample. Both the thickness and circular surface area were measured from optical microscope images processed using Image J software. These measurements were subject to varying degree of human error. However, the general trends shown here support the proposed hypothesis.

**Figure 2:**
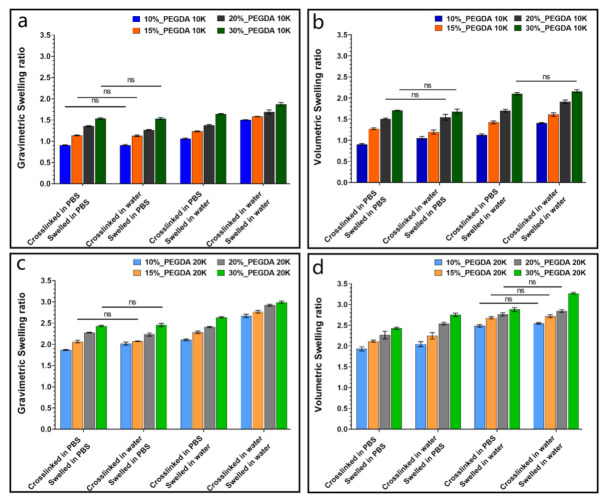
Gravimetric and volumetric equilibrium swelling ratio of PEGDA hydrogels crosslinked and swollen in different media. (**a**,**b**) 10k PEGDA with different polymer weight fraction (*n* = 5); (**c**,**d**) 20k PEGDA with different polymer weight fraction (*n* = 5). Groups without significant difference are labeled; otherwise, significant differences were present), (ns *p* > 0.05).

It is known that the swelling behavior of hydrogels is influenced not only by the polymer fraction and properties but also the environmental factors such as temperature, ionic strength, and pH [41]. For better understanding of how different factors affect the swelling ratio of neutral PEGDA hydrogels, different network composition and crosslinking and swelling conditions were studied. Figure 2 shows the influence of PEGDA weight fraction, starting molecular weight, and ionic strength of the medium on the swelling properties of both 10k and 20k PEGDA hydrogels. Compared to polyelectrolyte hydrogels, the swelling of neutral hydrogels such as PEGDA gels is less significantly influenced by ionic strength. However, ionic strength was considered an important factor, since different biological environments present varying ionic compositions. PBS was used in this study to simulate a representative biological environment. It is known that dissolved salts have a profound effect on the organization of water molecules. The dissolved anions and cations have a shell of water molecules around them—a so-called hydration shell—which acts to disrupt the intermolecular structure of the surrounding bulk water [44]. Similarly, the presence of ions would be expected to affect the equilibrium swelling of the hydrogels. 

**Figure 3:**
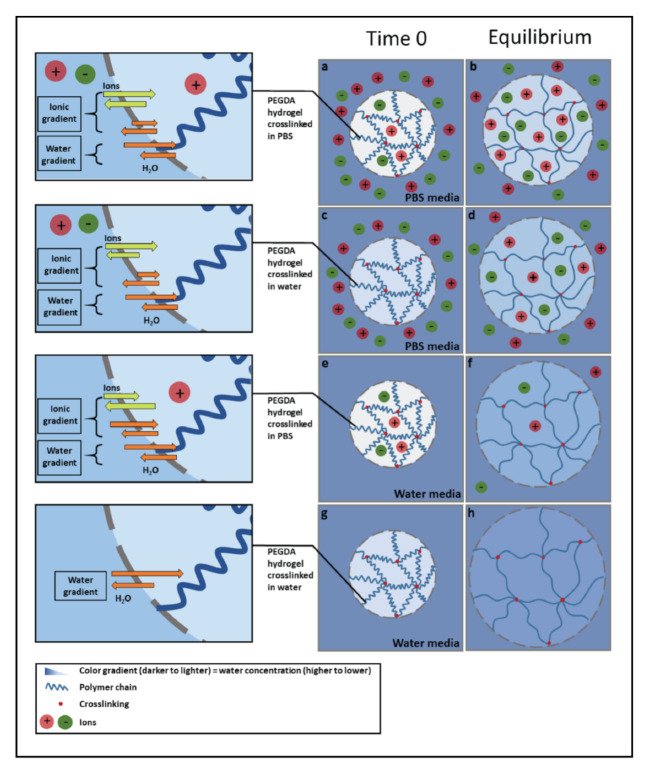
Graphic illustration of the mechanism of swelling of PEGDA hydrogel crosslinked and swollen in different media at time 0, and at equilibrium. (**a**,**b**) Crosslinked in phosphate-buffered saline (PBS), swollen in PBS; (**c**,**d**) crosslinked in water, swollen in PBS; (**e**,**f**) crosslinked in PBS, swollen in water; (**g**,**h**) crosslinked in water, swollen in water.

The neutral gels studied here displayed a considerable sensitivity to the ionic strength of the surrounding medium (Figure 2).Such behavior was ascribed to the presence of an ionic gradient between hydrogel and external medium, according to the proposed mechanism presented in Figure 3, which is described below. All hydrogel formulations showed an increased swelling ratio as the number of ions present in the hydrogel at equilibrium decreased (left to right in each individual panel of Figure 2). Figure 3 explains this effect schematically for the different combinations of crosslinking and swelling media. The extent of swelling at equilibrium depends on the net osmotic pressure due to the balance between the pressure imparted by the water gradient and the ionic gradient. Both water and ions can diffuse into and out of the gel to varying degrees, depending on this balance. The system that was both crosslinked and swollen in PBS would be expected to swell overall the least, because the net inward osmotic gradient is lowest for these gels. Gels crosslinked in water and swollen in PBS experience a significant outward pressure due to the higher ionic strength of the surrounding media. Gels both crosslinked and swollen in water experience only the inward pressure due to the presence of the polymer network and therefore have the highest equilibrium swelling ratio.

The equilibrium swelling ratio of the PEGDA hydrogels increased with higher molecular weight for the same weight fraction of polymer and swelling conditions (Figure 2a,b compared to Figure 2c,d). This was ascribed to the larger mesh size of the higher molecular weight hydrogels due to the greater distance between crosslinking points. Similarly, within the polymer weight fraction range of 10 to 30%, the equilibrium swelling ratio increased with increasing weight fraction for the same polymer molecular weight and swelling conditions. The increased concentration of the macromolecular network results in a corresponding amplification of the inward osmotic pressure due to a higher water gradient.

### 3.2. Thermal Analysis (DSC) and Water Content Determination

Differential scanning calorimetry (DSC) is commonly applied for studying different states of water in hydrogel systems [15]. Three such water states can be detected in hydrogels: (1) non-freezable bound water, which is strongly bound to the molecular chains of the polymer. It forms a primary hydration shell around the hydrophilic polymer chains by hydrogen bonding with the oxygen atoms of the PEGDA polymer; (2) freezable bound water, which forms hydrogen bonding with PEGDA similar to but weaker than non-freezable bound water. It forms the secondary hydration shell; (3) free water behaves similarly to bulk water, which scarcely interacts with the polymer surface [45,46,47]. The amount of water in each of these states observed for any hydrated polymer depends on the number and nature of the polar groups on the polymer as well as the amount of space between macromolecular chains (that is, the network mesh structure) and the elastic resistance of the network to deformation [48]. The amount of non-freezable bound water appears to be a fixed characteristic of a certain polymer composition (that is, for constant chain structure and network structure), since it is a function of the number of available polar sites for hydrogen bonding. For a given polymer structure, the amount of non-freezable bound water increases in a linear fashion up to a threshold, at which time all hydrogen bonding sites on the macromolecular chains are saturated. For subsequent increases in hydrogel water content, there is a negligible increase in the amount of non-freezable bound water, even at very high water content [48]. The amount of bound water has important implications for the hydrogel properties. For example, it has been shown using O^17^ NMR that the permeability to dissolved oxygen through the swollen polymer is the lowest when the amount of bound or “hydration” water is at its highest [49].

A representative DSC thermogram for 30 wt % of 20k PEGDA is shown in Figure S3. The major overlapping endothermic peaks during the heating process represent the melting of freezable bound water in large pores and free water. The minor peak observed below 0 °C represents the melting of freezable bound water in small pores [41]. The exothermic peak around −20 °C during the cooling process represents the crystallization of freezable bound water and free water. The large enthalpy value of this “loop” means there is a large amount of freezable water content in this hydrogel [50].

**Figure 4:**
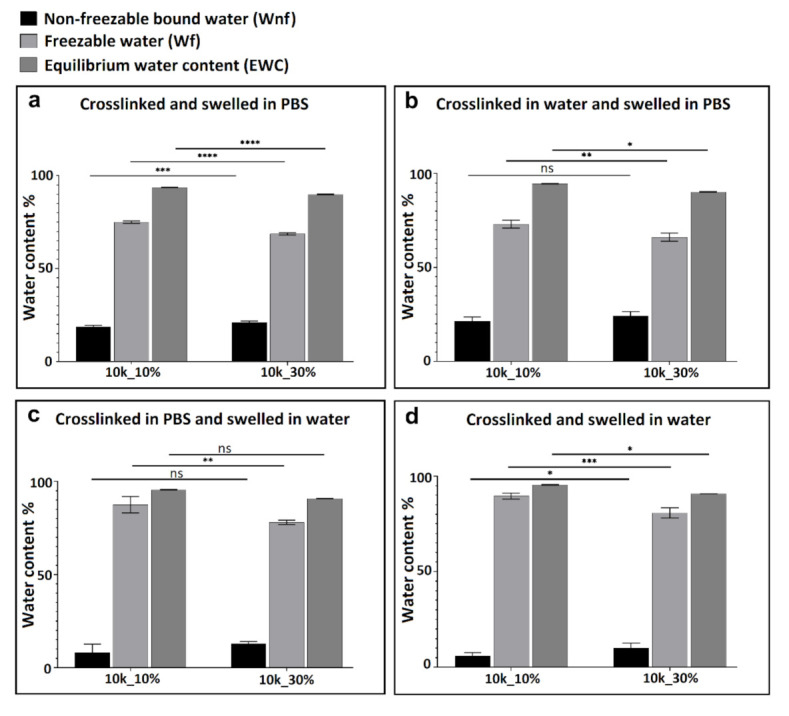
Water content for 10k PEGDA hydrogel. Comparison of 10 and 30% PEGDA weight fraction, crosslinked and swollen in different media (*n* = 3), (ns *p* > 0.05, * *p* < 0.05, ** *p* < 0.01, *** *p* < 0.001).

**Figure 5:**
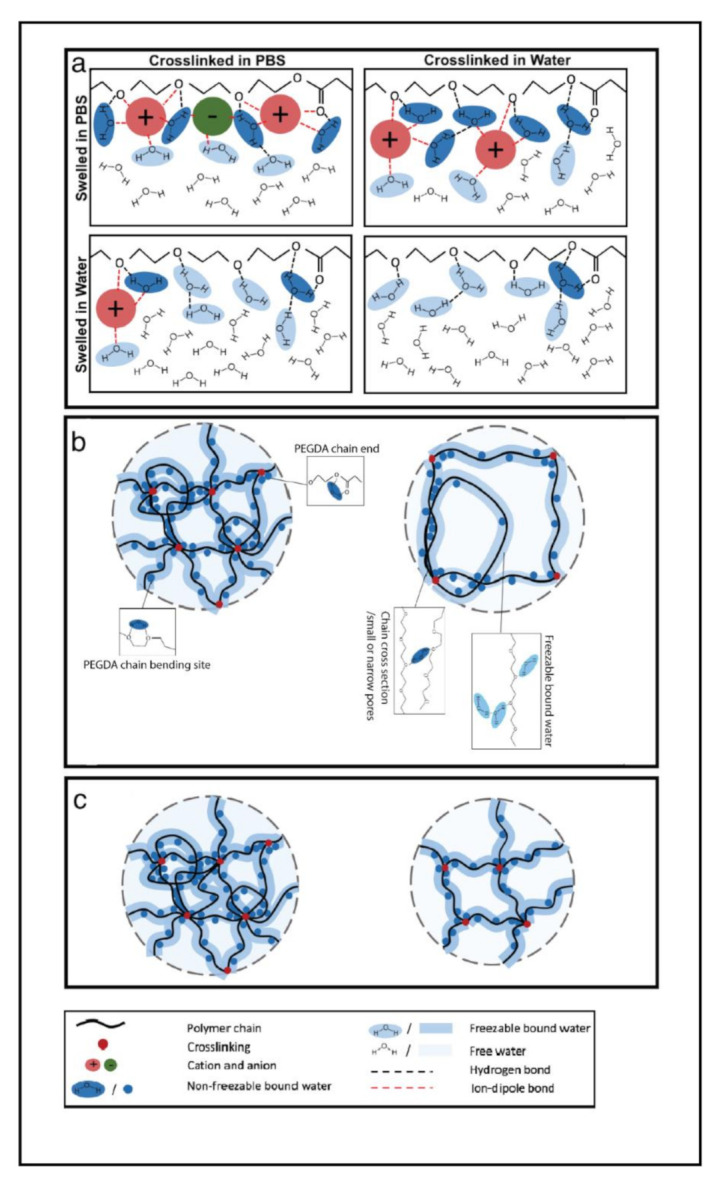
Graphic illustration of water content in PEGDA hydrogels. (**a**) Water content under different swelling conditions; (**b**) comparison of water content in hydrogels with different PEGDA molecular weight; (**c**) comparison of water content in hydrogels with different PEGDA weight fraction.

As described previously [15], the amount of freezable (*Wf*) and non-freezable (*Wnf*) water present in the hydrogel can be calculated from the DSC information using Equation (3), (4), and (5) (Experimental Section). This method was used here to quantitatively evaluate the influence of ionic strength of the swelling media, polymer molecular weight, and polymer weight fraction on the different types of water present in the neutral PEGDA hydrogels after swelling. As in the study of swelling ratio described above, hydrogels were crosslinked and swollen in different media (ultrapure water or PBS). The DSC experiments were performed to illuminate the mechanism of chemical interaction of water and ions with PEGDA polymer chains. Figure 4 summarizes the influence of polymer weight fraction on the different types of water content. Since the melting peaks of freezable bound and free water are overlapping, the sum of the enthalpy for all three endotherms was calculated and represents the total freezable water content (*Wf*). By comparing sub-figures a to d in Figure 4, it can be seen that regardless of crosslinking/swelling conditions and polymer molecular weight, non-freezable water content (*Wnf*) increased with increasing weight fraction of PEGDA. This is due to increasing the contact area between polymer and water, as illustrated in Figure 5c.

**Figure 6:**
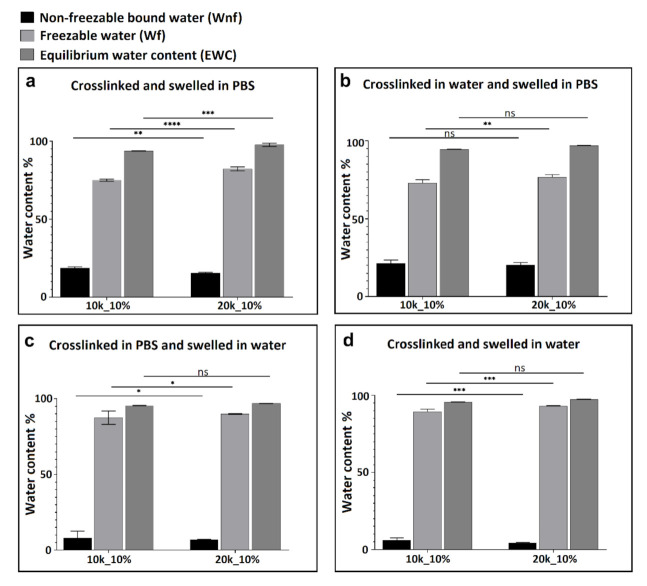
Water content for hydrogel with 10% PEGDA weight fraction. Comparison of 10 and 20k PEGDA, crosslinked and swollen in different media (*n* = 3), (ns *p* > 0.05, * *p* < 0.05, ** *p* < 0.01, *** *p* < 0.001).

Figure 6 shows 10 wt % PEGDA used as an example to illustrate the influence of PEGDA molecular weight on water content. By comparing the four sub-figures, regardless of crosslinking and swelling conditions, the non-freezable water content (*Wnf*) increased with decreasing molecular weight of PEGDA starting polymer. This greater tendency to form non-freezable bound water for lower molecular weight PEGDA is illustrated in Figure 5b. As the molecular weight decreases, the network density necessarily increases (smaller mesh size), decreasing the overall swelling due to elastic resistance but increasing the amount of bound water, which is most likely due to the increased proximity of the polymer chains, enabling strong hydrogen bonds between a single water molecule and two different polymer oxygen atoms. Another contributing factor could be the higher number of ester groups (from acrylate) for lower molecular weight PEGDA, which can form strong hydrogen bonds with water molecules. Figure 5b shows three proposed hydrogen bonding configurations of bound water on hydrated PEGDA chains [15]. Nonetheless, the influence of both molecular weight and PEGDA weight fraction on water content is only slight, and indeed, Figure 4b,c and Figure 6b all show no significant differences in percentage of non-freezable bound water (*Wnf*).

**Figure 7:**
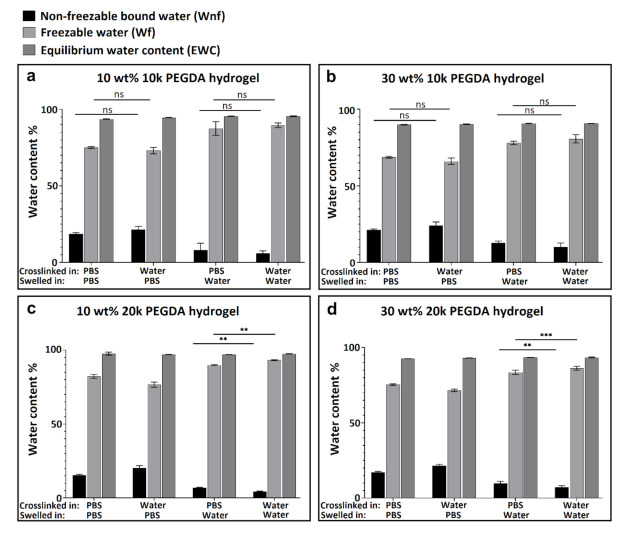
Water content for 10 and 20k PEGDA hydrogels with 10 or 30% PEGDA weight fraction. Comparison of crosslinking and swelling in different media (*n* = 3), (ns *p* > 0.05, ** *p* < 0.01, *** *p* < 0.001).

The presence of ions affects the balance of bound, intermediate, and free water in the hydrogels. Figure 7 shows how ionic strength in the system affected water content in the hydrogel after swelling. By comparing each sub-figure, regardless of molecular weight and weight fraction of PEGDA polymer, gels swollen in PBS showed more non-freezable water (Wnf) than those swollen in pure water. No significant differences were found between groups which were crosslinked in different media as long as the swelling media was kept the same, implying that the swelling media is more important than crosslinking media in determining the final water content. Based on the results, a conclusion can be drawn that the non-freezable bound water content (Wnf) increased with increasing ions in the swelling media. It is hypothesized that this behavior may be ascribed to increasing ion-dipole bond formation between cations in PBS and both water molecules and the oxygen atoms on the PEGDA polymer chain. This effect is illustrated schematically in Figure 5a. For hydrogels crosslinked in PBS, ion–dipole interactions could be foundational in the formation of the initial hydration layer. For samples subsequently also swollen in PBS, this layer remains intact; however, when the swelling medium is water, some of the ion–dipole bonds are displaced by hydrogen bonds of water molecules with the macromolecular polymer network. This may be due either to a simple concentration effect and/or differing binding affinities of the different species. For hydrogels crosslinked in water and subsequently swollen in PBS, the ions may be unable to displace many of the water molecules bound in the primary hydration layer during crosslinking.

The results presented here have given important insights into the interaction of water within hydrogels; however, it is possible to probe this phenomenon still further by considering the situation quantitatively at the molecular level. The number of bound water molecules per PEG repeat unit can be calculated and used to indicate the hydration properties and water-binding affinity in a hydrogel system [50]. The number of bound water molecules per PEG repeat unit has been estimated by a variety of techniques, including light scattering, NMR, and DSC, to be between one and four for linear, water-soluble PEG [51,52,53]. For linear PEG in concentrated solution, it is known that the maximum number of water molecules per PEG unit is molecular weight dependent, ranging from 3.3 to 2.4 to 1.6 as the polymer molecular weight decreases through the series 70,000, 1540, and 400, respectively [51]. Limited research has been conducted for determining the number of bound water molecules per repeat unit in a neutral hydrogel.

**Figure 8:**
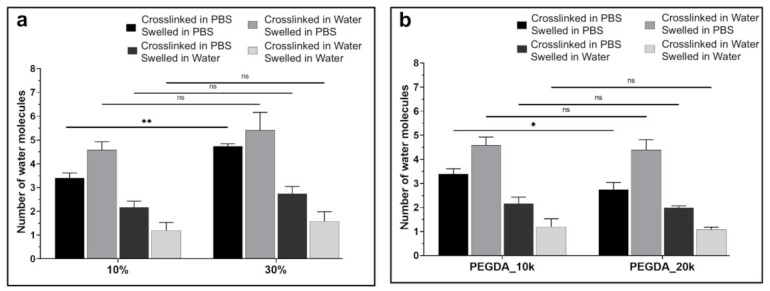
Number of non-freezable *bound water* molecules (*Nnf*) per repeating polymer unit for (**a**) PEGDA 10k with weight fraction of 10 or 30%, under different crosslinking and swelling conditions (*n* = 3); (**b**) 10% PEGDA weight fraction with molecular weight of 10 or 20k, under different crosslinking and swelling conditions (*n* = 3), (ns *p* > 0.05, * *p* < 0.05, ** *p* < 0.01).

The number of non-freezable bound (*Nnf*) water molecules per polymer repeat unit in hydrated polymers can be calculated from DSC data using Equations (6) and (7) (Experimental Section), as previously described [48,54]. Here, the results are presented in Figure 8 for *Nnf* in the swollen PEGDA hydrogels under different swelling conditions, with variable PEGDA weight fraction and molecular weight. Interestingly, no significant differences were found between groups having different weight fraction or molecular weight of PEGDA, indicating that although these factors affected overall swelling, they had little or no effect on bound water at the molecular level. This is not inconsistent with the results of Figure 4, Figure 6 and Figure 7, as it may first appear. Although non-freezable bound water content (*Wnf*) increased slightly with decreasing molecular weight (Figure 6), according to the proposed hypothesis, this was due to stronger binding by hydrogen bonding of individual water molecules between two separate polymer chains. This “shared” arrangement would result in an increase in overall non-freezable water but not necessarily any significant increase in the number of molecules per PEG repeat unit. When the crosslinking and swelling media were both water (effectively no ions present in the system), *Nnf* was the lowest and was between 1.2 and 1.6. The hydrogels that were swollen in PBS had the highest *Nnf*, between 2.8 and 5.5, which was likely due to the increased effective binding affinity as a result of strong ion–dipole interactions, as described in the swelling section.

Each PEG unit, having one oxygen atom, can hydrogen bond directly to two water molecules. The fact that up to six water molecules bound per PEG unit has been observed here is in line with the previously published conclusion that non-freezable water consists of not only water molecules in the initial hydration layer [48] (that is, directly hydrogen bonded to the polymer chain) but also water molecules hydrogen bonded indirectly via other water molecules or ion–dipole interactions. This theory considered together with the present results would suggest that the bound water of hydrogels swollen in the absence of ions consists of a single molecular layer of water, whereas in the presence of ions, the bound water comprises multiple layers of water molecules. The results are also consistent with those of Zhao et al., who studied a PEG-containing hydrogel swollen in PBS and with an EWC in a similar range to that presented here, and they found approximately six water molecules bound per PEG repeat unit [54]. This method of quantifying the amount of bound water in a hydrogel is of importance in assessing hydrogel systems for various applications, since it allows quantitative comparison between material systems or devices on a molecular scale, irrespective of size, dimension, or any other macroscopic characteristic. Further studies may elucidate specific correlations between these bound water parameters and different measures of bio-interaction, such as protein absorption.

Although the proposed model has several shortcomings, it can demonstrate the substantial changes experienced by hydrogels residing in an osmotic environment. This study has important implications beyond hydrogels. Most biological interactions with biomaterials are driven by direct contact of the material surface with the organism. It has been suggested that since a biomaterial comes in contact with water before it encounters proteins or cells, and similarly, a cell or protein comes in contact with the surface water before it contacts the material itself, the nature of the surface water layer may be the primary factor driving the interaction of materials with biological systems [1].

## 4. Conclusions

In conclusion, the dynamic swelling properties along with quantification of the different states of water content for PEGDA hydrogels were investigated. The influence of PEGDA weight fraction, molecular weight, and ionic strength of the medium on swelling properties and water content was studied. Both the gravimetric and volumetric swelling ratio increased with increasing molecular weight and weight fraction of PEGDA and decreasing ionic strength of the swelling media due to a combination of network effects and the shifting balance of osmotic pressure. DSC analysis showed a high freezable water content (*Wf*) in the hydrogels, with the remaining water content accounted for by non-freezable bound water (*Wnf*), which forms the primary hydration shell around the PEGDA polymer chains. The non-freezable bound water content was found to increase with increasing PEGDA weight fraction and ionic strength of the swelling media, and with decreasing PEGDA molecular weight. These results can be explained by variable dynamic hydrogen bonding and ion–dipole interactions between water molecules, ions, and the polymer chains. The molecular nature of these interactions could be further investigated using complementary techniques such as NMR and FTIR. The modulation of water states and composition is expected to have important implications for the biological response to the hydrogels [11], and further study into this is warranted. For example, quantitative protein adsorption and cell adhesion onto the surface of biomaterials could be directly studied as a function of the bound water content parameters studied here.

## Data Availability

Data is contained within the article and supplementary information.

## Supplementary Materials

The following are available online at https://www.mdpi.com/2073-4360/13/6/845/s1, Figure S1, Figure S2, Figure S3, Table S1: Hydrogel composition and media conditions for crosslinking and swelling.
