# Supplementary material for: Elucidating the Molecular Mechanisms for the Interaction of Water with Polyethylene Glycol-Based Hydrogels: Influence of Ionic Strength and Gel Network Structure
Source: Polymers (Basel). 2021 Mar 10;13(6):845. doi: 10.3390/polym13060845 (PMC8000404; doi:10.3390/polym13060845)
Supplement: Supplementary file 1 [file polymers-13-00845-s001.pdf]

polymers

Supporting Information

Elucidating the Molecular Mechanisms for the Interaction of Water with Polyethylene Glycol-based Hydrogels: Influence of Ionic Strength and Gel Network Structure

Xin Yang, Bronwin L. Dargaville and Dietmar W. Hutmacher *

Centre for Transformative Biomimetics in Bioengineering, Queensland University of Technology, 60 Musk Avenue, Kelvin Grove QLD 4059, Australia, x48.yang@hdr.qut.edu.au (X.Y.); bronwin.dargaville@qut.edu.au (B.L.D.)

* Correspondence: Dietmar.hutmacher@qut.edu.au

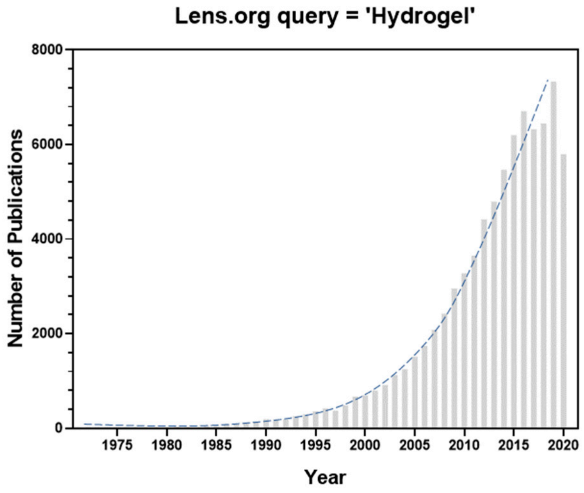

(A)

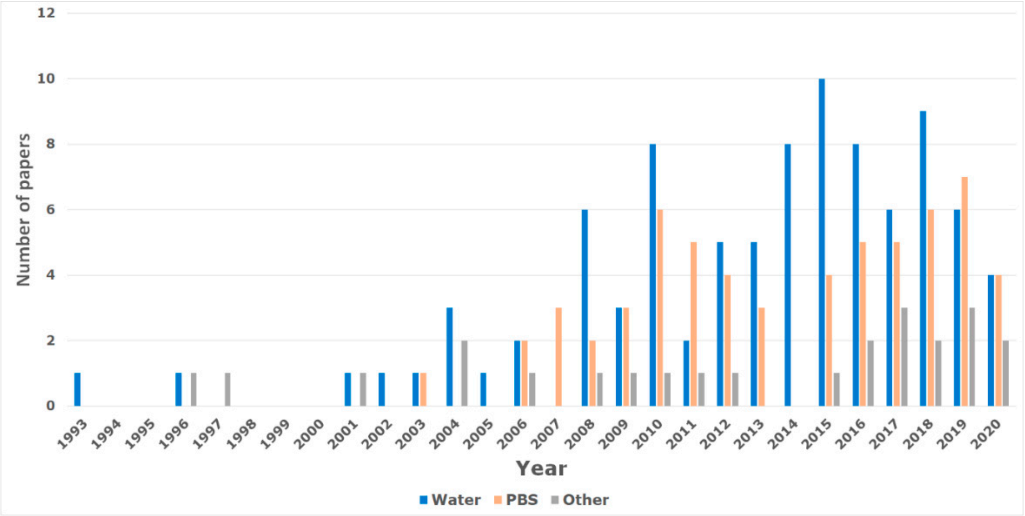

(B)

Figure S1. A. Number of publications related to the keyword "hydrogel" since the nineteen sixties. **B.** Published papers on the topic of swelling of PEGDA hydrogels from 1993 to the present. Papers focussing on swelling in water, PBS or other media are displayed separately. Data source: Lens.org**.**

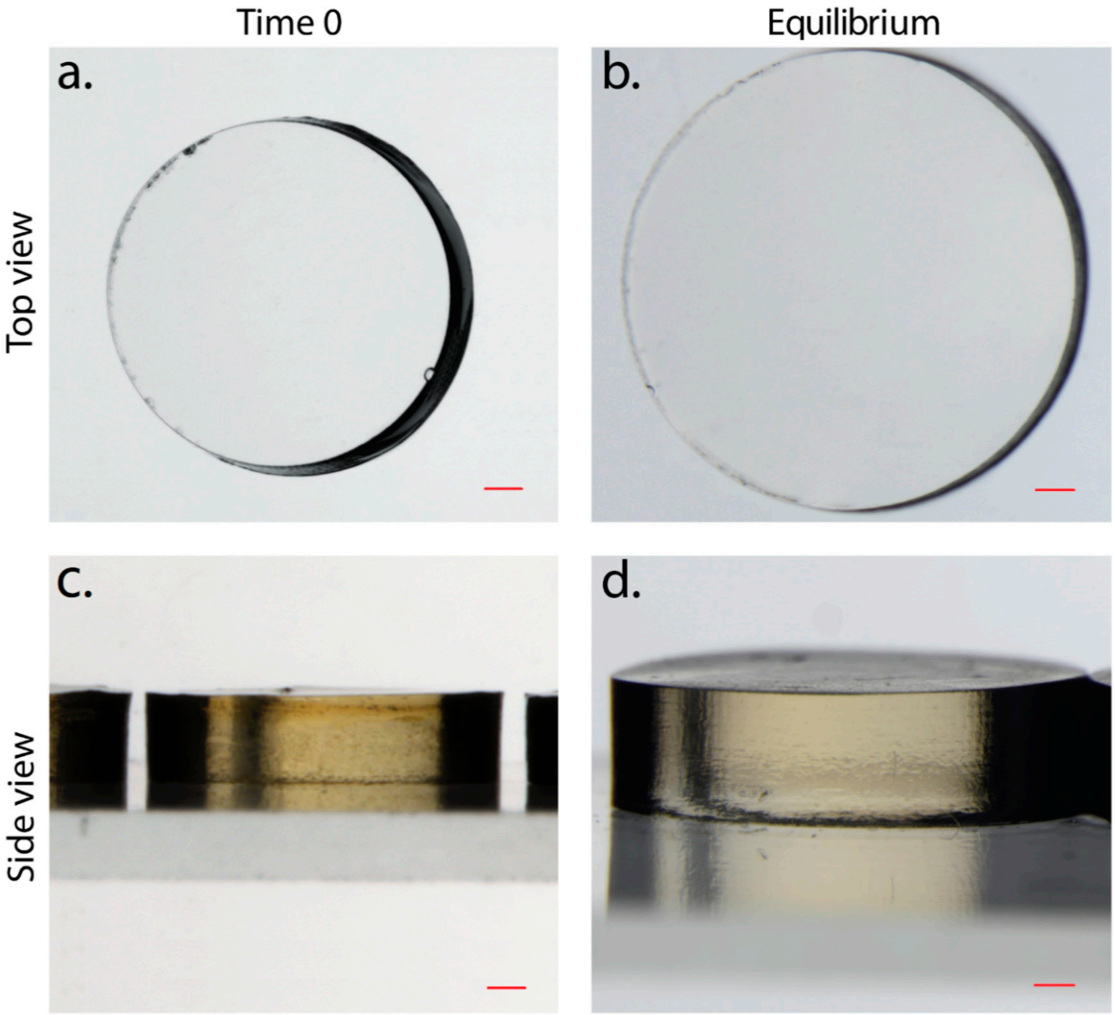

Figure S2. Optical microscopy photos of initial (**a**,**c**) and equilibrium state (**b**,**d**) of PEGDA 10 k, 20 wt%, crosslinked and swelled in ultrapure water.

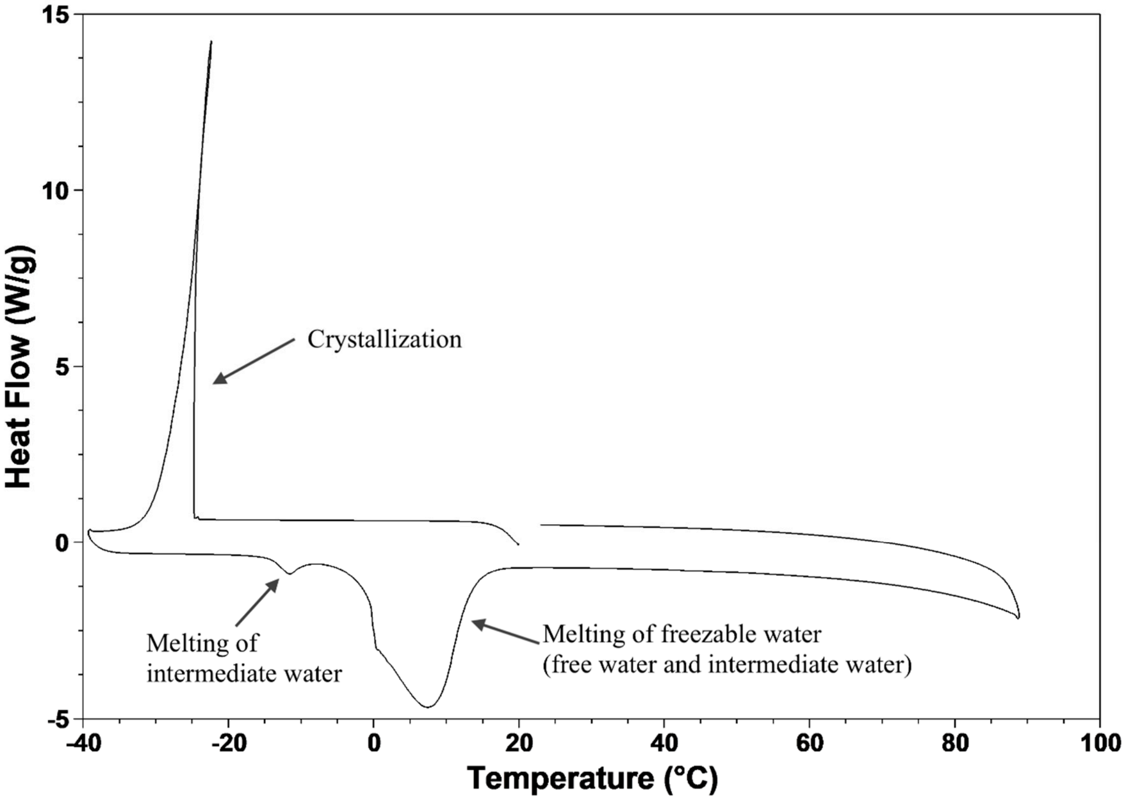

Figure S3. Differential scanning calorimetry (DSC) thermogram of 30% 20 k PEGDA crosslinked in PBS and swelled in water.

Table 1. Hydrogel composition and media conditions for crosslinking and swelling (n = 5).

**Polymer**	**PEGDA (10 k or 20 k)**															
Conc.of PEGDA (wt/wt) (%)	10	15	20	30	10	15	20	30	10	15	20	30	10	15	20	30
Crosslinking solvent	PBS				Ultra-pure water				PBS				Ultra-pure water			
Swelling medium	PBS				PBS				Ultra-pure water				Ultra-pure water			
